# Monitoring Pest Insect Traps by Means of Low-Power Image Sensor Technologies

**DOI:** 10.3390/s121115801

**Published:** 2012-11-13

**Authors:** Otoniel López, Miguel Martinez Rach, Hector Migallon, Manuel P. Malumbres, Alberto Bonastre, Juan J. Serrano

**Affiliations:** 1 Department of Physics and Computer Architecturer, Miguel Hernandez University, Ave. Universidad s/n-Ed. Alcudia, 03202 Elche, Spain; E-Mails: otoniel@umh.es (O.L.); mmrach@umh.es (M.M.R.); hmigallon@umh.es (H.M.); 2 Department of Computer Engineering, Technical University of Valencia, Camino de Vera s/n, 46021 Valencia, Spain; E-Mails: bonastre@disca.upv.es (A.B.); jserrano@itaca.upv.es (J.J.S.)

**Keywords:** pest detection, trap monitoring, image processing, image sensors, low power devices

## Abstract

Monitoring pest insect populations is currently a key issue in agriculture and forestry protection. At the farm level, human operators typically must perform periodical surveys of the traps disseminated through the field. This is a labor-, time- and cost-consuming activity, in particular for large plantations or large forestry areas, so it would be of great advantage to have an affordable system capable of doing this task automatically in an accurate and a more efficient way. This paper proposes an autonomous monitoring system based on a low-cost image sensor that it is able to capture and send images of the trap contents to a remote control station with the periodicity demanded by the trapping application. Our autonomous monitoring system will be able to cover large areas with very low energy consumption. This issue would be the main key point in our study; since the operational live of the overall monitoring system should be extended to months of continuous operation without any kind of maintenance (*i.e.*, battery replacement). The images delivered by image sensors would be time-stamped and processed in the control station to get the number of individuals found at each trap. All the information would be conveniently stored at the control station, and accessible via Internet by means of available network services at control station (WiFi, WiMax, 3G/4G, *etc.*).

## 1. Introduction

Integrated pest management relies on the accuracy of pest population monitoring techniques. Without gathering information about the population dynamics together with the related ecological factors it is almost impossible to execute the appropriate pest control at the right time in the right place [1,2]. Ecological factors in the environment can be classified as physical (e.g., temperature and humidity), chemical (e.g., chemical composition of the soil), and biological factors (e.g., pathogens and pests). Among these factors, pests are those that directly damage the crop, and pest control has always been considered the most difficult challenge to overcome.

A well-known technique to perform pest control monitoring is based on the use of insect traps conveniently spread over the specified control area. Depending on the kind of insect, each trap is properly installed with pheromones or other chemical substances that attract the insect we want to capture. The traps are designed in such a way that insects entering in the trap are unable to leave it, so pest monitoring systems will periodically collect the data of each trap (captured individuals count) to perform an efficient pest control monitoring.

The popular method to collect trap data consists on repeated field surveys where visual observation of traps is performed by a human operator to record the number of captured insects. The periodicity between two consecutive surveys is usually between 15 to 30 days. This method has two main drawbacks: (1) it is labor intensive and therefore costly and (2) all monitoring traps cannot be synchronized to measure the target pest population. Given that the traditional monitoring techniques are labor intensive and offer poor temporal resolution measurement, the dynamics of pest population density in the field cannot be accurately monitored. Consequently, a proper estimation for a target pest population will be limited to a long-term scale.

These are the main reasons that justify the use of image sensor network technologies to perform automatic pest monitoring. Therefore, we will propose in this paper a low-cost system based on battery-powered wireless image sensors, that accurately monitor pest populations with a higher temporal resolution and a significant reduction of pest monitoring costs, as no human intervention is required during the monitoring process.

There are a lot of wireless image sensor proposals in the literature that may be used in our target application, so we will provide a representative view of the available wireless image sensors platforms, in particular of the Imote2 [3], developed by the Intel Corporation and being manufactured by MEMSIC. It is an advanced platform especially designed for sensor network applications requiring high CPU/DSP and wireless link performance and reliability. The Imote2 contains an Intel XScale processor, PXA271, with an IEEE 802.15.4 radio (TI CC2420) and one onboard antenna. It has 256 KB SRAM, 32 MB Flash, and 32 MB SDRAM, and provides basic and advanced expansion connectors supporting: 3xUART, I2C, 2xSPI, SDIO, I2S, AC97, USB host, Camera I/F, GPIO. A mini USB port is also available to establish connection with a PC. The size of an Imote2 is 48 mm × 36 mm.

The CMUCam3 [4] is an ARM7TDMI based fully programmable embedded computer sensor. It is equipped with a Philips LPC2106 processor that is connected to an Omnivision CMOS camera sensor module. Features of CMUCam3 include: CIF resolution (352 × 288 pixels) RGB color sensor, open source development environment for Windows and Linux, and an MMC flash slot with FAT16 driver support able to store captured video into memory at 26 frames/s. It contains LUA (a lightweight scripting programming language) for rapid prototyping, software-based JPEG compression, basic image manipulation library, CMUcam3 image emulation, compatible connector with wireless motes (Tmote Sky, FireFly, 802.15.4), and FIFO image buffer for multiple pass hi-resolution image processing. It supports 64 KB RAM and 128 KB of ROM.

MeshEye [5] is a hybrid resolution smart camera mote for applications in distributed intelligence surveillance. MeshEye has a low resolution stereo vision system that continuously determines the position, range, and size of moving objects entering in its field of view. This information triggers a color camera module to acquire a high resolution image of the object. It contains an ARM7TDMI thumb processor. It is a 32 bit RISC architecture that can operate at up to 47.92 MHz. MeshEye has 64 KB of SRAM and 256 KB of flash memory. It uses a CC2420 radio transceiver and provides an implementation of the IEEE 802.15.4 standard. It uses the Agilent 2700 VGA camera module.

In [6], a vision-enabled image processing framework for sensor motes is presented. It uses FireFly motes coupled with CMUCam3 sensor cameras. The main design objective of FireFly Mosaic is to design a low cost, energy efficient, and scalable camera mote compared to the centralized wireless webcam-based solutions. FireFly Mosaic runs Nano-RK, a real time operating system for WSNs and it uses the networking protocol stack provided by Nano-RK. FireFly motes, CMUCam3, and Nano-RK are developed at Carnegie Mellon University.

The Fleck hardware platform [7] is designed for low-bandwidth wireless camera networks where image compression is undertaken at each node. Fleck is a robust hardware platform for outdoor use. The current version of FleckTM-3 consists of an ATmega 128 microcontroller running at 8 MHz. It is equipped with a Nordic NRF905 radio transceiver with a bit rate of 76.8 Kbits/s. Another promising feature of Fleck is that it is equipped with solar charging circuitry. It provides an additional DSP board which is a Texas Instruments 32 bit 150 MHz DSP processor (TMS320F2812). It has 1 MB of SRAM and an Omnivision 640 × 480 pixel color CCD sensor.

Most of the wireless image sensors listed above are intended for general purpose wireless sensor network applications, some of them are designed with powerful processors and a wide set of I/O devices/modules that in practice require considerable power demands, so their autonomy is really constrained. Others are designed for specific applications so they cannot be easily reused for other applications like the one we are interested on. For this reason, we also review some proposals focused on our target application: pest trap monitoring systems based on image processing capabilities.

In [8,9] the authors propose an innovative decision support system for *in situ* early pest detection based on video analysis and scene interpretation to detect the presence of certain insects inside a greenhouse. Several wireless video cameras are suitably installed in the greenhouse capturing HD video at 10 frames per second. The video is recorded through a motion detector sensor that triggers video recording only when there are insects in the camera visual field. The captured video is delivered to a central server where video analysis algorithms combined with a priori knowledge about the visual appearance of insects (e.g., shape, size, color) are applied to the incoming video sequences to detect them. This approach is very ambitious since it was designed to monitoring different kinds of insects, but its cost is too high, requiring also a lot of communication and computing resources.

In [10] a monitoring system designed to monitor the fruit fly pest is proposed. The sensor is just a smartphone that includes on-board camera and native cellular communication capabilities. In this work, the sensor nodes periodically capture one image and deliver it to the central server through 3G data service available at trap location. Image processing is performed in the central server to detect the presence and number of fruit flies in each trap. The authors show results using only one trap, but there are two main limitations to this idea: (1) image sensor cost related to the own terminal and the cost of each image upload through 3G data service, and (2) the power consumption demanded by the smartphone which is solved by authors with the use of solar panels, although there is no further details about it.

In [11] the authors propose a remote pest monitoring system based on traps equipped with image sensors that wirelessly reports traps pest status inside a greenhouse. This work is the closest one to our proposal. It is based on low-cost image sensor nodes and wireless communication support to deliver the captured images of trap contents to a central station, where image processing determines the number of captured individuals. It is restricted to a greenhouse area for detecting the number of trapped insects but without identifying them. Also, the power consumption is not optimized being the image sensor operational live limited to 34 h, as authors state in their experimental study. Although this would not be a serious problem in a greenhouse (limited area) where daily maintenance can be performed, in other applications like the ones we are interested on (tens of traps distributed over large areas) would be a great constraint due to the high maintenance costs derived from battery replacement. This question shows the importance of optimizing power consumption in the image sensor design.

Consequently, we propose a pest monitoring system that, like similar works in the literature, will provide several benefits over the traditional manpowered trap monitoring process: (1) it works in an unattended mode, (2) it is able to significantly reduce the monitoring costs, (3) the temporal resolution of trap monitoring data is higher and may be programmable, and (4) trap monitoring data may be available in real time through an Internet connection.

Our proposal is able to perform an efficient pest monitoring process based on the design of appropriate wireless image sensors distributed over the monitoring area. The main advantages with respect its competitors (described above) are: (1) higher scalability, being able to deploy in small monitoring areas (greenhouses) as in large plantation extensions, (2) low-cost wireless image sensor (less than 100 € per sensor), (3) low-power consumption that allows zero maintenance during the operational life of nodes.

Although our proposal could be used for monitoring several kinds of pests, our work is mainly focused on the Red Palm Weevil (RPW, *Rhynchophorus ferrugineus* (Olivier)) pest that actually it is present in a large number of regions around the world attacking different species of palm trees (*i.e.*, date palm, coconut palm, and royal palm).

**Figure 1.**
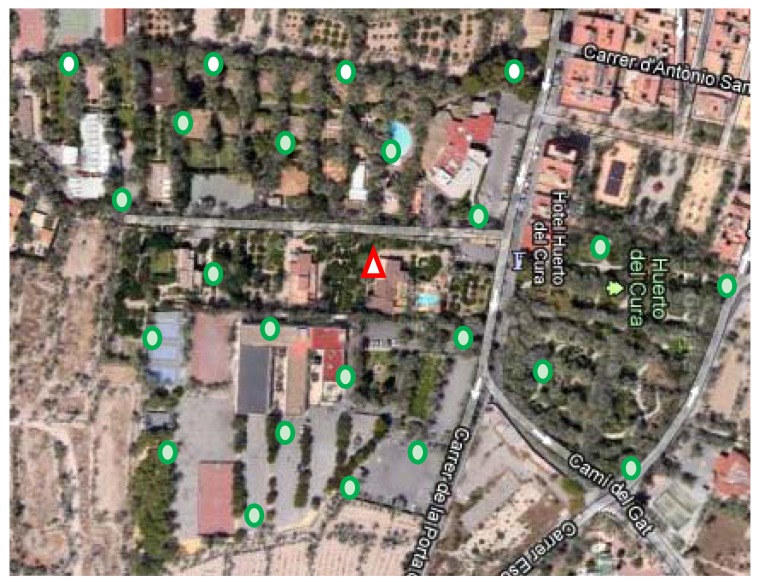
An example of trap deployment. Each circle represents a trap equipped with wireless image sensor. The triangle in the middle represents the location of the control station.

Therefore, our pest monitoring proposal is based on RPW traps equipped with wireless image sensors that are scattered over the monitoring area forming a Wireless Image Sensor Network (WISN). In Figure 1, we show an example of WISN deployment. Each trap, represented by a green circle, is equipped with a wireless image sensor that periodically takes a picture of trap contents that it is delivered to the control station. In Figure 1, control station is represented by a red triangle. Control station deals with (1) the storage of the captured images delivered by the image sensors, and (2) the processing of the captured images in order to determine the number of individuals found at each trap by means of image recognition algorithms like the one proposed in [12], that it is able to identify RPW individuals with a success rate higher that 95%. All the captured images with the corresponding metadata (timestamp, GPS location, processing results, *etc.*) are conveniently stored in the control station. Also, this information is accessible in real time by means of internet service providers available at the control station location (WiFi, WiMAX, 3G/4G, ADSL, Cable, Satellite, *etc*.).

The overall RPW pest monitoring project is comprised of several development stages: (1) the design and implementation of a low-cost wireless image sensor prototype, (2) the field experimental testing by means of trap deployment in a reduced monitoring area, and (3) based on the picture set gathered in the field testing stage, we will develop and test the image recognition software that accurately provide the number of RPW individuals present in each picture. In this paper we present the results of the first developing stage, the design and implementation of a wireless image sensor prototype.

## 2. Image Sensor: System Description

In this section we will provide details about the design of our proposed image sensor, showing the components of the sensor node architecture and describing its operating software. As explained before each image sensor will be installed inside a trap.

**Figure 2.**
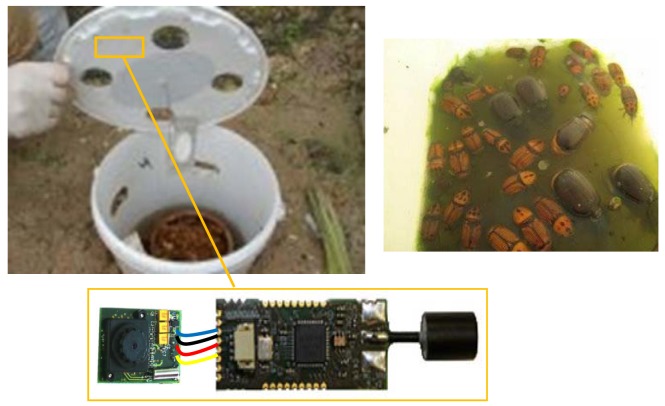
An example of one RPW trap, a snapshot of trap contents, and our wireless image sensor prototype.

In Figure 2, we show a trap example specially designed for RPW pest monitoring and a snapshot of trap contents where several RPW individuals were captured. The proposed image sensor will be located inside the trap, fixed at the top and facing the onboard camera towards the bottom of the trap where the RPW captured individuals will remain. It is important to provide enough illumination inside the trap, so a translucent plastic material is recommended for the trap. Also, by simply providing some holes at the top should be enough to guarantee proper lighting conditions. The size of the image sensor (batteries included) is 60 mm × 35 mm × 20 mm, being small enough to be placed in any trap design.

Traps equipped with image sensors are distributed across the monitoring area in such a way that each sensor may contact with at least another one (connected network) and so forming a Wireless Sensor Network (WSN). In this way, if a particular image sensor is in the radio coverage of the central station, its captured images may be directly delivered to the control station (single hop communication), otherwise the captured images should be delivery through intermediate image sensors to reach central station. The WSN topology, the way each sensor node is connected with other network nodes, typically follows a mesh pattern in order to get regular distributed measurements over the whole monitoring area. This would provide an interesting property for our pest monitoring application: high error resilience since, in mesh topologies, each node has more than one available neighbor in its radio coverage area (*i.e.*, neighborhood degree greater than 1), so in case one sensor fails (hardware error or battery depletion), the network remains connected, since its neighbor nodes may reach the central station bordering failed node.

In this work we will focus in the image sensor design and operation, so in order to simplify our study we will suppose all image sensor nodes are in the radio coverage of central station (single hop communication). Despite of this, more complex topologies, such as tree-clustered organization, have been successfully deployed in other projects, and would be also applicable to this environment.

### 2.1. Hardware Design

Sensor nodes typically capture scalar data values like temperature, pressure, humidity, *etc.*, but in our case the captured data is represented by an image, so the device complexity is an issue here: each sensor node should contain a camera, a radio transceiver and a microcontroller with enough computing power to manage image capture and transfer processes. Although there are a bunch of devices that can be used for our target application, we have designed and developed a low-power sensor node based on the CC1110F32 SoC from Texas Instruments [13]. The CC1110F32 is a true low-power sub-1 GHz system-on-chip (SoC) designed for low-power wireless applications. It combines the excellent performance of the state-of-the-art RF transceiver CC1101 with an industry-standard enhanced 8051 MCU, with 32 KB of in-system programmable flash memory and 4 KB of RAM, and many other powerful features. The small 6 × 6 mm package makes it very suited for applications with size limitations. The CC1110F32 is highly suited for systems where very low power consumption is required. This is ensured by several advanced low-power operating modes what provides a very promising feature for our long-life pest monitoring system.

**Figure 3.**
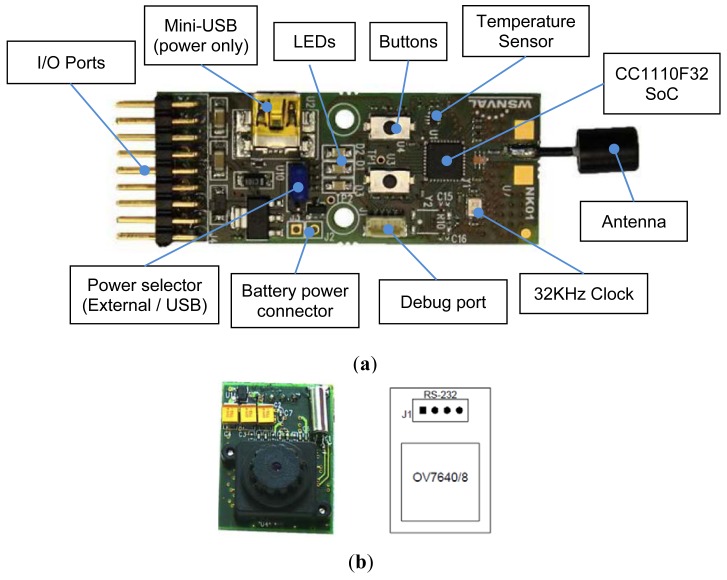
Image sensor design: (**a**) wireless sensor board based on CC1110F32 SoC, and (**b**) camera module connected to sensor board through USART I/O ports.

In Figure 3(a) we show the main components of the wireless sensor architecture, where we can find the CC1110F32 SoC that includes a 8051 enhanced microcontroller unit (MCU), the CC1101 radio transceiver module, and a set of peripherals such as 128-bit AES security coprocessor, one USB 2.0 interface, two USARTs (working also in SPI mode), one I^2^S interface, three 8 bit timers, one 16 bit timer, 7–12 bits ADC, and 21 GPIO pins.

The CC1101 radio transceiver was configured to work in the 868 MHz band. There are different modulations available that allows data rates up to 500 Kbps. The effective radio coverage may change depending on (1) several parameters that directly depend on the trap location, as terrain profile, radio wave obstacles, noise interference levels at the selected transmission channel and trap location (at ground level or fixed at the palm tree at an specific elevation over the ground), (2) the transmission power level of CC1101 radio (up to 10 dBm), and (3) the antenna type employed [13].

We have performed some outdoor experiments to determine the radio coverage of our image sensor in Line Of Sight (LOS) environments similar to the one shown in Figure 1. At the highest data rate (250 Kbps), with the standard antenna and the maximum available transmission power (10 dBm) we have established point-to-point communications over distances of up to 500 m. We have fixed the maximum point-to-point communications distance in 300 m, where the received signal strength is about −81 dBm, just 10 dB above CC1101 radio receiver sensitivity threshold [12] and the observed average packet error rate was around 1%. Reducing the transmission power to the default value (0 dBm), the one used in all of our experiments, the maximum distance is reduced to 140 m.

As mentioned above, our study assumes a single-hop communication scenario where every node should be in the coverage area of control station. So, all sensor nodes should be up to 140 m away from the control station location, which roughly represents a 10,000 m^2^ monitoring area. As future work, we plan to evaluate other network scenarios (multi-hop, relay based, *etc.*) to increase the overall area coverage of our pest monitoring proposal.

The choice of the camera was also critical. It should have high resolution, low cost and low power consumption. We have chosen a low-cost C328-7640 color camera from Comedia [14], shown at Figure 3(b). It is able to produce raw or JPEG-compressed images [15] at a maximum resolution of 640 × 480 pixels provided by the OmniVision OV7640 image sensor. The camera is connected with the sensor board through a UART-standard serial port that it can manage data rates up to 115 Kbps. The camera module requires only 60 mA in full operation and it also has available a power standby mode to significantly reduce power consumption at sensor node sleep periods. We use the USART0 interface to connect sensor board with the camera module trough the I/O corresponding pins. The USART0 was configured with an 8N1 character format and a bit rate of 115,200 bps.

Finally, we would like to supply some details about the proposed battery unit. In addition to nominal voltage and capacity, the choice of the battery depends on the ambient conditions and the shelf life of the device. Since we consider non-rechargeable batteries, we chose a lithium thionyl chloride (Li-SOCl_2_) battery. This battery technology offers high energy density, operating voltage stable during most of the application lifetime, wide temperature range (−55 °C to 85 °C), performance independent from cell orientation and long shelf life and reliability (over 10 years). In particular the chosen reference was the ER14250 battery from EEMB or EVE, for example. The main characteristics are nominal voltage equal to 3.6 V and capacity equal to 1,200 mAh. The battery is a cylindrical cell of size 14.5 × 25.2 mm, according to the device size.

### 2.2. Operating Software

The software is stored in the on-chip flash memory (32 Kbytes) and is loaded at boot time. Just after hardware reset or power on, the sensor node enters in the initialization stage. If it is properly configured, announces its availability through a HELLO broadcast control message before passing to the next stage. If it is not configured (first boot), the sensor node enters in configuration mode waiting for instructions from control station (CS). If control station does not receive the HELLO message, then it delivers to sensor node the configuration information like node ID, GPS location, sensor node hardware address, image format (resolution, color or B&W, *etc.*), image capture period and the schedule for the first image capture. After receiving configuration data: (1) it configures sensor board (wireless radio and peripherals), then (2) broadcast the HELLO message indicating the end of the configuration mode, and finally (3) the sensor node enters in standby mode to save power until the first scheduled image capture begins.

In practice, the initialization mode is done only once, when the trap is installed in the field. During the image sensor installation, the human operator will install the trap into the desired monitoring location by means of the trap deployment software running in a portable device (smartphone, tablet, laptop, *etc.*) with GPS support and equipped with the control station radio interface (USB dongle) which allows direct communication with the image sensor under installation. The trap deployment system will act as the control station during the initialization stage, without requiring the synchronization with other network nodes. The deployment system will consider that the trap is installed and waiting for the first capture cycle just after receiving the HELLO message. After installing all the traps, the information recorded by the deployment system is downloaded to the control station, so it will know all the information related to the installed image sensors (ID, hardware address, GPS location, capture cycle schedule, *etc.*).

**Figure 4.**
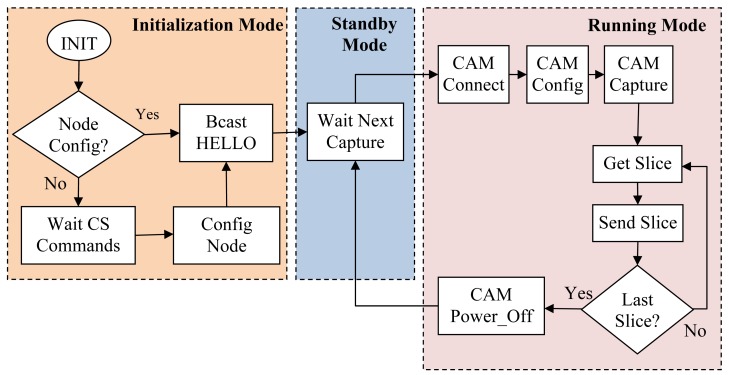
Flow diagram of the image sensor operating software, with its three differentiated working modes: Initialization, standby and running.

In Figure 4, we show, by means of a flow diagram, the different stages of the operating software installed in each image sensor node. After initialization stage, the sensor node enters in a loop of running and standby modes defined by the image capture period established in the node configuration. So, at the beginning of a capture period the sensor node enters in running mode, gets one image and delivers it to the central station. Once the image is sent, the sensor node enters in standby mode waiting until the beginning of the next capture cycle. In our study we will employ capture cycles that range from 30 min to one day.

In running mode, the first operation that sensor node should done is power on the camera module and establish the communication between sensor board and camera. Then, it configures the camera with the desired picture parameters (Image resolution, JPEG compression, *etc.*), and finally it takes a snapshot. After that, a pipelined image delivery process is launched where sensor node first downloads a picture slice (fragment of the captured picture) from camera and then delivers it to the control station as payload of a wireless data packet. The packet size is a critical configuration parameter since it determines de total number of packets that a compressed image will produce in the delivery process. So, if network constraints are not considered here (*i.e.*, bit error rate), we will define a packet size as large as possible taking into account the limitations of the available memory required for buffering issues. Finally, we fixed the maximum packet payload length in 240 bytes. Once the last image slice is delivered to control station, the sensor node switches to standby mode, remaining in this state until the next capture cycle starts again.

## 3. Image Sensor: Power Consumption Analysis

After describing the proposed image sensor design and coding the operating software, we will proceed to perform a detailed power consumption analysis in order to estimate its autonomy with standard battery cells. For that purpose, first we will perform a timing profile of the basic operations executed in the running mode using different image resolutions. Then, we will perform a set of power consumption measurements of sensor node, to determine the required energy in standby and running modes. In running mode we will measure the power consumption of every single basic operation, to determine the most energy-intensive operations. Finally, taking in mind the power consumption analysis we will estimate the image sensor autonomy with standard battery units, varying the image size (80 × 64, 160 × 128, 320 × 240, 640 × 480) and the image capture period (0.5, 1, 3, 6, 12 and 24 h between two consecutive images).

### 3.1. Timing Profile for Running Mode Basic Operations

We have measured the time required to capture and deliver one image with each of the available image sizes. For that purpose, we have used the high precision 16 bit timer to perform accurate time measurements (microsecond resolution). All time measurements were repeated 100 times providing the average value with a 95% confidence interval. As previously shown at Figure 4, running mode is composed of six basic steps described as follows:

✓ CAM_Connect: It powers on the camera and establishes connection through the USART0 port.
✓ CAM_Config: Once the camera is connected and ready, this process configures the camera with the proper image parameters (image size, capture mode, *etc.*).
✓ CAM_Capture: This operation sends the capture command to the camera. After capturing the image in its internal memory, the camera performs JPEG compression with a fixed quality level.
✓ T_Get_Slice: It represents the downloading process of the compressed image from camera. It is performed in chunks we called image slices. As mentioned before, the size of each slice is fixed at 240 bytes, with the exception of the last one. This operation quantifies the total time required to download all the image slices from camera.
✓ T_Send_Slice: This process is related with the wireless transmission to the control station of all slices belonging to the captured image. This operation is interlaced with the previous one, so after downloading one slice (Get_Slice) from camera, this process starts its wireless transmission (Send_Slice;. So, it represents the total time required to deliver all the image slices.
✓ CAM_Power_Off: Before exit running mode, the camera is powered off to save power.

**Figure 5.**
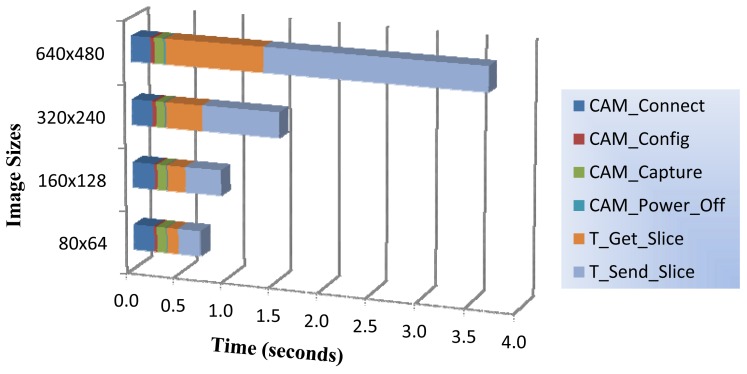
Timing profile of running mode operations.

In Figure 5, we show the latency of the running mode operations. As it can be seen, the time spent on receiving the sequence of slices corresponding to the compressed image (T_Get_Slice), and sending them to the central station (T_Send_Slice) represents a significant portion of the total time. In particular, T_Send_Slice takes 32.17% of the total time for the smallest image size (about 5 slices on average) and 62.18% for the largest image size (about 40 slices on average). Take into account that the number of packets (slices) resulting from the JPEG compression for a particular image format size, will depend on the characteristics and features of the captured image content (textures, details, *etc.*).

Notice that operations not devoted to managing image slices like initialization, configuration, capture and image compression, are independent of the selected image size (all of them are completed in 0.365 s approx.).

### 3.2. Image Sensor: Power Consumption Profile

After the timing profile of running mode operations, we will perform electric current measurements of both standby and running modes. We have also measured the current demanded by our image sensor node when it is in standby mode, being a constant current flow of 8 μA. The camera module showed an average consumption of 63.6 mA at nominal operation, and the power required by radio transmitter and receiver is about 16 mA and 18 mA, respectively. The CC1110F32 SoC consumes an average of 5.6 mA when working in running mode (radio off).

Now, we perform a set of electric current measurements (one measure every 2 ms) during the execution of each basic operation to calculate the average value. After that, for each basic operation we can determine the power consumption in mAh units, as the average duration of each operation and its demanded electric current are known.

**Figure 6.**
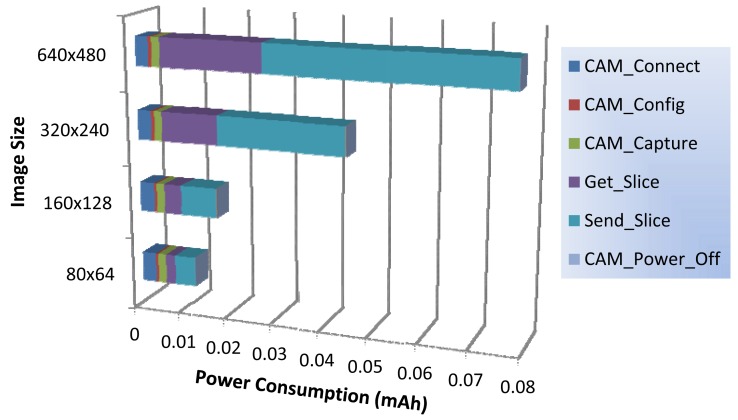
Power consumption profile of the different running mode operations.

In Figure 6 we show the power consumption profile of running mode operations. As with the timing profile, we can observe that those operations not involved with slice data management have near constant power consumption for all image sizes. The slice management operations are the ones that most power demands, exponentially increasing the required power as the image size increases. In particular, the transmission of the image slices (T_Send_Slice) represents the 36%, 46%, 61% and 66% of the overall power consumption for 80 × 64, 160 × 128, 320 × 240, and 640 × 480 image sizes, respectively.

This fact shows us that much of the energy consumed in the running mode will largely depend on the number of slices we have to send via radio to the central station. Thus, we think that the image compression system should play a fundamental role in reducing energy consumption, and as a consequence it would allow us to further extend the lifetime of our node. We will back to this issue in a later analysis.

### 3.3. Image Sensor Autonomy

With the results obtained before, we may estimate the autonomy of our image sensor equipped with standard battery units, varying the image size (80 × 64, 160 × 128, 320 × 240, and 640 × 480) and the image capture period (0.5, 1, 3, 6, 12 and 24 h between two consecutive images).

**Figure 7.**
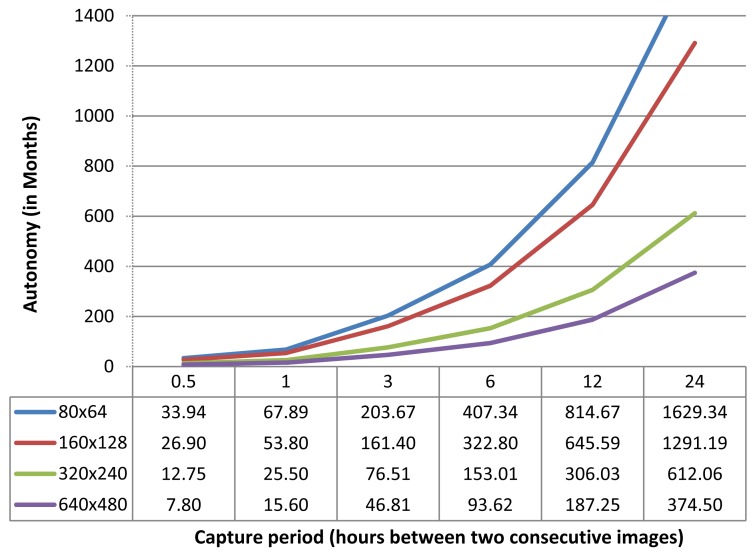
Autonomy of our image sensor equipped with 1,200 mAh battery (80% efficiency) with different image format sizes and capture frequencies.

In Figure 7, we show the autonomy of the image sensor node powered with the proposed battery unit with a nominal capacity of 1,200 mAh (assuming a battery efficiency of 80%) at different image format sizes and image capture periods. As explained before, the image sensor is in standby mode most of the time with ultra-low power consumption (∼8 μA). When the capture cycle starts, the sensor node moves to an active state to capture, compress and send the image before returning to standby mode until next capturing cycle. As it can be observed there is room for usability, since for the largest image format size (640 × 480 YUV 4:2.1) and the highest capture frequency (1 image every 30 min) the node autonomy is estimated in more than seven months. At the other hand, node autonomy is increased up to more than 25 years (in this case, the sensor node autonomy is limited by the battery operational life time. The proposed batteries are designed to work between 10 to 15 years at optimum operating parameters (temperature, current discharge, *etc.*)) using the same image format size but with a longer capture cycle of one image at day.

Remember that we have made this study assuming a direct communication between sensors and central station (single hop network), and we do not care about other network related issues, as packet error delivery. Although single hop communication approach would be enough for trap deployment in greenhouses or small monitoring areas, the support for network delivery errors should be taken into account. This would not represent any additional complexity in terms of sensor node resources (memory or computing power), but it will increase the power consumption of the image sensor. So, depending on the environmental noise present at the monitoring area, we will suffer a particular packet error rate. This would imply the retransmission of the image slices that were lost with the corresponding power consumption overhead.

**Figure 8.**
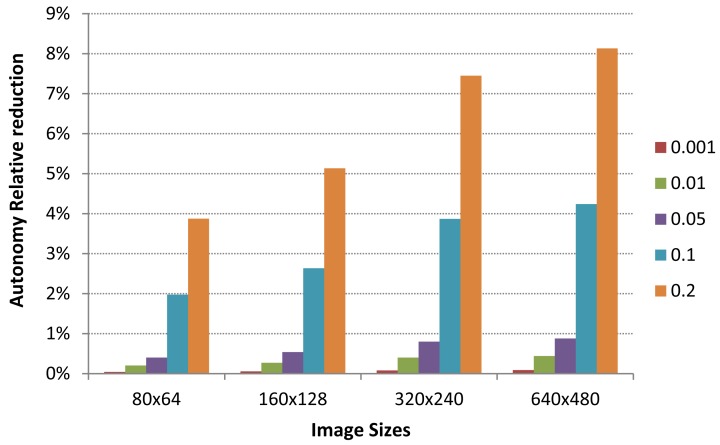
Autonomy relative reduction due to packet delivery errors. Results are given for each available image size working at maximum capture frequency (1 image every 30 min) and different packet error rates (from 1 out of 1,000 up to 1 out of 5).

To illustrate the impact of packet transmission errors in power consumption, we show in Figure 8 an approximation of the image sensor autonomy reduction at different packet error rates (0.001, 0.01, 0.05, 0.1 and 0.2) working at the highest capture frequency (one image every 30 min). With the 640 × 480 image size, the captured and compressed images are fragmented in an average of 49 slices. So, in order to deliver the whole image to the central station we need to transmit 49 packets. Depending on the actual packet error rate, some of these packets may require retransmission due to channel errors, increasing the total number of packets that effectively were transmitted to central station. This issue leads to an increase of power required to transmit each captured image in a more realistic network conditions.

As it can be seen in Figure 8, the autonomy of our image sensor is reduced as the packet error rate grows. However, the autonomy reduction under extreme channel errors conditions (one packet out of five is lost or with errors) is around 8% for the highest image size. Considering the image sensor autonomy shown at Figure 7, we conclude that the impact of packet delivery errors over autonomy is very limited for regular packet error rates.

## 4. Improvements to Reduce Power Consumption

From the power consumption profile, we have observed that image slice management operations, downloading image slices from camera and their subsequent wireless transmission, are the ones that most energy demands, being up to 90% of the overall sensor energy consumption. Thus, in order to improve the energy consumption of our image sensor node we should work over these operations.

On the one hand, the image wireless transmission is the operation that most energy demands, being in some cases more than 60% of the overall energy consumed. So, improving the performance of the image compression system, we will significantly reduce the energy consumption of the wireless image delivery operation, and as a consequence the overall energy consumption. On the other hand, downloading the image from camera module also requires a lot of energy (up to 30% of the image sensor energy consumption), so one approach to save energy is to enhance the data transfer between camera and sensor board by means of a faster interface that greatly reduce the image transfer time.

Therefore, improving image management operations should lead to a significant reduction of power consumption that would allow us to extend the lifetime of our image sensor node, or seen it in another way, for a specific node lifetime we could use the saved energy on other complementary tasks (*i.e.*, powerful software compressors) or in additional hardware/software functionality that improves image sensor node (*i.e.*, higher resolution image sensors).

To implement these improvements we need to: (1) replace the actual camera module by another one with similar characteristics but with a faster communication interface, and (2) implement a fast and efficient image encoder that may run under the resource constrained CC1110F32 SoC platform.

The new camera module proposed is the C329-SPI, considered as the next version of the C328-7640 with the same low-power consumption profile, available image resolutions and board dimensions. The most important difference is the available SPI communication interface, allowing data rates up to 24 Mbps (200 times faster than the employed USART transfer rate). It also includes a JPEG hardware compressor with three quality levels, and near the same command interface than its predecessor. It is interesting to note that compression level should be carefully addressed since high compression rates degrade the quality of reconstructed image. So, we have to take care of compression level in such a way that it does not disturb the image analysis process to identify the captured insects. This process will depend of the morphology of target insects. In our scenario, we have to detect RPW weevils with sizes around 3 cm long and a characteristic shape and color. As the size of target insect becomes lower, the compression noise will become a serious problem for the detection algorithm. So, in these situations, we have to find the highest compression level that does not interfere in the detection success rate.

With respect to the software image compressor, we propose a line-based image compression scheme, since its working mode perfectly matches with the way the camera sends the captured image to the sensor node (line by line in a raster order). Thus, we do not need to store the entire raw image in the sensor memory before starting the compression process. With an image line-based encoder, we read the image lines directly from the camera to encode and transmit them on-the-fly. This encoder will be composed by a line-based 2D-DWT transform module, a uniform scalar quantizer, and a fast run-length encoder [16].

It demands very low memory amounts which will be proportional to the image size and the length of the wavelet filter. The line-based 2D-DWT transform performs a 5-level dyadic decomposition by means of a convolution with an integer-based bi-orthogonal 5/3 filter bank. As explained in [15], the line-based 2D-DWT transform would require the storage of no more than 2 · (2*N* + 1) · *w* 16 bit integers, where N is fixed by the filter length (Bi5/3 → N = 2) and *w* is the image line width. So, with the largest image size (640 × 480 → w = 640), the required memory for line-based 2D-DWT would be 12,800 bytes (In [9], we propose a lifting line-based 2D-DWT algorithm, which would require near the half of memory than the convolution version. However, for coding simplicity we decide to use convolution version as enough memory is available). Both quantization and run-length encoding also demand some buffer storage to perform their duties. In particular, at each decomposition level we will require 4 rows of each subband (HL, LH, HH) to conform the encoder buffer, and this will sum up no more than 2 · (4 · 3) · *w*/2) integers (15,236 bytes for 640 × 480 image size).

However, although the extra memory needed is affordable, the computational power required by this encoder (one of the lowest computational demanding wavelet encoders) surpasses by large the capacity of our CC1110F32 SoC system. To prove that, we implemented in our image sensor node the 1D-DWT transform (convolution approach) to get timing measures that allow us to estimate the time required to perform the full 5-level 2D-DWT wavelet decomposition.

**Figure 9.**
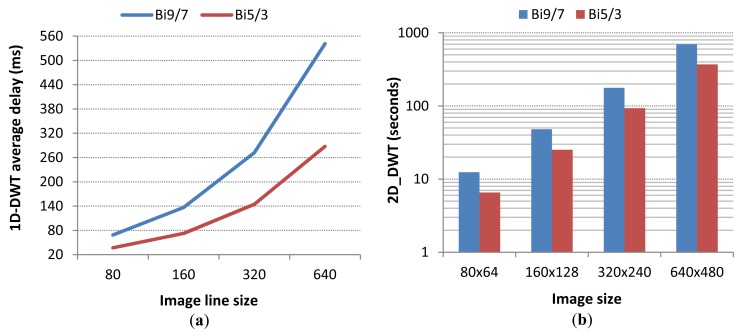
DWT complexity using Bi9/7 and Bi5/3 filter banks: (**a**) 1D-DWT computing time, and (**b**) estimation of the 2D-DWT execution time (logscale).

As it can be seen in Figure 9(b), the time required to perform the 2D-DWT transform is not shorter than 6 s for the lowest image resolution, being up to more than 500 s for the highest image size. Although the code may be optimized, the 2D-DWT complexity is unaffordable for CC1110F32 architecture.

So, at the moment, the only improvement we may apply to our design is the faster SPI communication interface between sensor node and camera. For that purpose, the operating software was modified at the running mode stage (see Figure 4) in order to include SPI master-slave interface driver (simpler than the one we have coded for the asynchronous USART management) running with master clock rate set at 3 MHz. The basic operations of running mode will be the same with the exception of “Get_Slice”, that now will be a faster operation due to SPI interface, and “CAM_Connect” that would not require the camera synchronization process being simpler and faster than the older one.

Respect to the timing profile performed in Section 3.1, we would assume that basic operations will have the same timings with the exception of the ones that were modified: CAM_Connect and Get_Slice. The first one would be significantly shorter since synchronization is avoided thanks to the SPI synchronous interface. In particular, the sensor node requires an average of 25 SYNC command exchange through the USART interface to receive the ACK command that declares synchronized the connection, so we may estimate the timing of the new CAM_Connect operation by simply deducing the time wasted in synchronization (an average of 190 ms) from the timing obtained in Section 3.1 (218.2 ms, in average). The last modified operation, Get_Slice, is much faster than the one defined with the initial image sensor design, as downloading one slice of 240 bytes trough SPI connection interface will considerably reduce the overall transfer time, specially at the largest image sizes.

**Figure 10.**
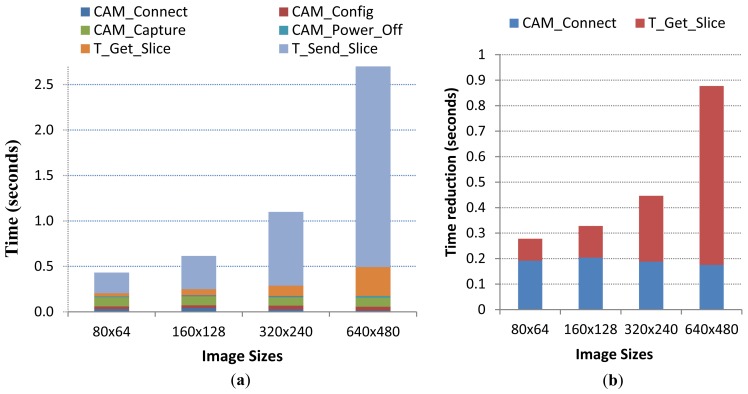
(**a**) Running mode timing profile with the new SPI camera, and (**b**) the absolute time reductions found at CAM_Connect and T_Get_Slice operations for each image size.

In Figure 10(a), we show the new timing profile of the running mode stage, where basic operations CAM_Connect and T_Get_Slice are the only affected by the SPI camera improvements. As explained before, the rest of basic operations have the same timings than the ones obtained in Section 3.1. So, as it can be observed, we were able to greatly reduce the download time of the compressed slices from camera (up to 700 ms in the largest image size), and also to reduce the time spent to establish camera connection (190 ms in average). These improvements have reduced the running mode execution time in all image sizes, as shown in Figure 10(b), being up to 0.9 s for the highest image size. This would also imply a reduction in power consumption and as consequence an enlargement of the image sensor operational life. After adjusting the new power consumptions, the resulting autonomy of our image sensor with the new SPI-Camera is extended in a 25% for all image sizes. So, configuring the image sensor node to take pictures at 640 × 480 size every 30 minutes and the same batteries than the ones described in Section 3.3, the image sensor autonomy increases from 7.8 to 9.8 months.

## 5. Conclusions

Monitoring pest insect populations is a fundamental issue in agriculture and forestry protection. One of the most popular methods to monitoring pests is by means of a set of traps strategically distributed across the desired monitoring area. To collect trap data, typically, human operators visually check the traps and annotate the number of captured individuals with a frequency of 15 to 30 days. This method has two main drawbacks: (1) it is labor intensive and therefore costly and (2) all monitoring traps cannot be synchronized to measure the target pest population. Given that the traditional monitoring techniques are labor intensive with a poor temporal resolution measurement, the dynamics of pest population density in the field cannot be accurately monitored. Consequently, the estimation for a target pest population will be limited to a long-term scale.

In order to improve the trap monitoring pest system, we proposed a low-cost system based on battery-powered wireless image sensors, that accurately monitor pest population with a higher temporal resolution and a significant reduction of pest monitoring costs, as no human intervention is required during the monitoring process. The main advantages of our proposal are: (1) higher scalability, being able to deploy in small monitoring areas (greenhouses) as in large plantation extensions, (2) low-cost wireless image sensor (less than 100 € per sensor), (3) very low-power consumption that allows zero maintenance during the operational life of sensor nodes (up to 25 years), (4) temporal resolution of trap monitoring data is higher and it can be programmable, and (5) trap monitoring data may be available in real time through an Internet connection.

The proposed image sensor system may be easily fixed inside the existing traps to wirelessly deliver images to central station. When images are received at central station, typically a PC with no power restrictions, they may be processed applying image recognition algorithms to evaluate the number of individuals found at each image. The processing results are attached to each image as metadata as well as the timestamp and geolocation info, and the resulting images are conveniently stored in the control station, making them available through an internet connection.

Although this work is focused in the image sensor design, considering a simple communication model with the central station (direct communication), it proves that wireless image sensor technology will leverage the deployment of efficient pest monitoring systems with a considerably cost reduction (low-cost nodes and no human monitoring costs) and near zero maintenance due to the large operational life of the proposed image sensors.
